# The association between health enhancing physical activity and neighbourhood environment among Swedish adults – a population-based cross-sectional study

**DOI:** 10.1186/1479-5868-6-8

**Published:** 2009-02-09

**Authors:** Patrick Bergman, Andrej M Grjibovski, Maria Hagströmer, James F Sallis, Michael Sjöström

**Affiliations:** 1 Unit for Preventive Nutrition, Department of Biosciences and Nutrition, Karolinska Institutet, SE-14157 Huddinge, Sweden; 2 Norwegian Institute of Public Health, Postbox 4404, Nydalen, 0403 Oslo, Norway; 3 San Diego State University, Active Living Research, 3900 Fifth Avenue, Suite 310 San Diego, CA 92103, USA

## Abstract

**Background:**

This study examines the relationship of neighbourhood environment factors with walking and total health enhancing physical activity.

**Methods:**

A population-based cross-sectional study. The short self-administered version of the validated International Physical Activity Questionnaire (IPAQ) was used to assess health enhancing physical activity including walking. The neighbourhood environment was assessed using a 17-item environmental module. A principal component analysis among the environmental variables was conducted. The factor scores were divided into tertiles and independent associations between factor tertiles and physical activity categories and walking were studied by multinomial logistic regression with adjustment for confounders.

**Results:**

In adjusted models, a lower odds ratio (OR) for reaching the middle, OR: 0.66 (95% Confidence Interval (CI): 0.47–0.98), and upper, OR: 0.65 (0.45–0.95), tertile of walking was observed among those in the lowest tertile of the *degree of urbanisation*. A higher OR for reaching the middle, OR: 1.84 (1.28–1.64), and upper tertile, OR: 1.64 (1.14–2.36), of walking was observed among those in the lowest tertile of *fear of crime*. A higher OR for reaching the high category of total health enhancing physical activity was observed among those in the lowest, OR: 2.01 (1.32–3.05), and middle tertile, OR: 1.52 (1.02–2.25), of the factor *degree of urbanisation*.

**Conclusion:**

The findings suggest that the environment is differentially related to walking and total health enhancing physical activity. This should be explored in future research to disentangle the complex relationship between different levels and aspects of physical activity and their relationship with the environment.

## Introduction

To date, many interventions to promote physical activity have shown disappointing results, particularly with regard to the long-term maintenance [1]. More recent public health strategies to promote physical activity among populations acknowledge the role of the physical environment. Different ecological models of physical activity have been presented [2,3]. Common to all are that they focus on individual influences as well as on social and environmental factors that may facilitate or inhibit individual behaviour [4]. Ecological models hypothesize that several levels of influence determine physical activity-related behaviour. The levels of influence can broadly be categorised into intra-individual factors and extra-individual factors [3]. The intra-individual factors might include individual attributes such as, beliefs, attitudes and behaviours. The extra-individual factors may include physical environment, social and cultural contexts, and policy. This explicit emphasis on physical environmental factors, as potential influences on physical activity, is the main feature of the ecological models applied to physical activity. The physical environment is comprised of urban form, land use, the transportation system, recreational resources and green space [2,5,6]. The physical environment is believed to influence physical activity and therefore has the potential, through modification, to promote physical activity [7,8]. The advantage of this approach is that a modification of the physical environment is expected to have relatively permanent effects and to affect entire communities or populations [9].

However, research on the association between the physical environment and physical activity has several limitations. Firstly, around 80% of all research on the relationship between physical activity and the physical environment has been conducted in the USA and Australia [10]. The results from these studies are not easily translated to European, or Swedish, settings. Secondly, even if some large scale studies exist [11,12], the settings within which the association between physical activity and the physical environment have mainly been studied are relatively small, such as within one community [13], or a few neighbourhoods [14,15]. The large scale studies are also often limited in their crude assessment of the physical environment ("*The area where I live offers many opportunities to be physically active?*"). This reduces the environmental variability which may lead to underestimation of true associations with physical activity, and also the generalisability of the findings. Thirdly, total physical activity, which should be most strongly related to health outcomes, has usually not been reported. Most studies have only investigated the association between sub-components of physical activity predominantly walking and physical environment [15,16]. Although walking is an important contributor to total physical activity [17] and is associated with health outcomes [18], it may not be a good reflection of the total physical activity.

Therefore, it is of interest to public health to examine the association between the perception of the physical and social environment and levels of both walking and total physical activity in a large scale.

In this study we present data on the relationship between the perception of physical and social attributes of the local neighbourhood environment with both self-reported walking and total health enhancing physical activity in a nationally representative sample of Swedish adults.

## Methods

### Study design

This is a population-based cross-sectional study and it is a part of the International Physical Activity Prevalence Study (IPS). The IPS began in 2002 and is a worldwide collaboration between 20 countries. The aim with IPS is, using the International Physical Activity Questionnaire (IPAQ)  to obtain internationally-comparable physical activity prevalence estimates in a large scale pilot study. From the Swedish population 2500 individuals, of both genders, aged 18–74 years, were sampled at random from the national post and address registry. The short self-administered version of the IPAQ, with an additional environmental module, was the main instrument for studying health enhancing physical activity. The questionnaire was mailed to the subjects who returned it completed using a pre-paid return envelope. Consistent with the IPS protocol, all data were collected in October-November 2003. This study was approved by the research ethics committee at Huddinge University Hospital (432/03).

### Data collection

The short version of IPAQ estimates how much health enhancing physical activity a person has performed over the previous seven days and covers four domains of physical activity (at home, during transport, occupational and leisure time physical activity). The subjects estimated how many days (frequency) they were physically active and, on such days, the average time (duration) they spent physically active. This was done for physical activity of three intensities (vigorous physical activity, moderate physical activity and walking). The IPAQ short form has been shown to have acceptable test-retest reliability and criterion-related validity in a 12-country evaluation study that included Sweden [19].

The 17-item environmental module assesses the perception of physical and social attributes of the local neighbourhood environment, using a four-point Likert scale (agree, somewhat agree, somewhat disagree, disagree), with the exception of two questions. "*What is the main type of housing in your neighbourhood?*" for which the options were: Detached single-family or two family residences; row houses; apartments or condos of 1–2 stories; apartments or condos of 3–5 stories; apartments or condos of at least 5 stories. "*The number of motorised vehicles in working order in the household" *was an open-ended question. The environmental module was translated from English to Swedish, and relevant examples of environmental features were given. The local neighbourhood is defined as the area within a 15-minute walk from home. The questions addressed variables believed to be associated with recreational physical activity such as the presence and maintenance of foot and bike paths and recreational facilities. Environmental variables believed to be related to active transport included walking distance to shops, services, and public transportation, as well as access to motorised vehicles. The social environment was assessed by the perception of other physically active people in the neighbourhood, safety from traffic, and safety from crime. The environmental module has been shown to have acceptable test-retest reliability in a Swedish sample [20].

Questions on gender, age, income, education, employment status, marital status, smoking status, and self perceived health, as well as and height and weight, were also included in the questionnaire.

### Physical activity

The physical activity data were scored according to the 2005 revision of the IPAQ scoring protocol [21], with one exception. To avoid a large drop-out (approximately 25%), all subjects that in one or more intensity category had reported days (frequency) but not time (duration) of physical activity, or vice versa, were recoded as having spent zero time in that intensity category. To reduce the effect of known measurement errors [22-24] and to minimise the effect of the skewed data we divided the physical activity into categories (Table 1) in accordance with the IPAQ research committee and others [25]. The cut-off limits for the physical activity categories are based on the current guidelines for physical activity which state that every adult should be active on most, preferably all, days of the week, at moderate intensity, accumulating 30 minutes of daily physical activity [26]. In terms of how the IPAQ measures activity this would be equal to the cut off for the moderately active category or 600 MET minutes·week^-1 ^(5 days·30 minutes·4.0 MET) (1 MET equals the energy expenditure of sitting down quietly, 3.5 ml O_2_·kg^-1^·min^-1^).

**Table 1:** Physical activity categories and cut-off levels based on the IPAQ scoring protocol

Physical activity category		Cut off levels
1	Low	- no activity is reported or - some activity is reported but not enough to meet categories 2 or 3.
		
2	Moderate	- 3 or more days of vigorous activity for at least 20 min. per day or - 5 or more days of moderate intensity activity or walking for at least 30 min. per day or - 5 or more days of any combination of walking, moderate intensity or vigorous intensity activities achieving a minimum of 600 MET min·week^-1^
		
3	High	- 3 or more days of vigorous activity accumulating at least 1500 MET min·week^-1 ^or - 7 days of any combination of walking, moderate or vigorous intensity activities achieving a minimum of 3000 MET min·week^-1^.

See:

Walking was analysed separately because it is perhaps the most common type of physical activity. For the analyses on walking only, the total amount of reported time spent walking during the last seven days was divided into tertiles; low, (< 80 minutes), moderate (80–300 minutes) and high (> 300 minutes).

### Data presentation

Age was divided into three categories; 18 – 34 years, 35 – 54 years and 55 – 74 years. Body Mass Index (BMI) was calculated by dividing body weight in kilograms by height in meters squared (kg·m^-^^2^). BMI was recoded into three groups, normal (< 25), overweight (25 – 30) and as obese (> 30) [27]. Income was recoded into four groups; less than 100 000 Swedish Kronor/year (SEK/year), 100 000 to 200 000 SEK/year, 200 000 to 300 000 SEK/year and greater than 300 000 SEK/year (1 € ~10 SEK). Marital status was recoded into married/living with partner or single. Smoking status was recoded as one of three categories; current smoker, former smoker and non-smoker. Self-rated health originally had five categories (excellent, very good, good, satisfactory and poor) but due to small numbers in the two lowest categories (satisfactory and poor), they were combined into one.

### Data analysis

A one sample t-test and a Z-test were used to compare the mean age and gender structure, respectively, of the sample with corresponding characteristics of the adult population of Sweden [28]. Univariate associations between sample characteristics and categories of health enhancing physical activity were analysed by Pearson's χ^2 ^test of homogeneity. Group differences in median time spent walking by sample characteristics was analysed by Mann-Whitney U-tests or Kruskal-Wallis H-tests where appropriate.

Given that responses to many of the questions in the environmental module may be correlated, an exploratory principal component analysis (PCA) was performed to avoid multicollinearity and to reduce the number of variables by creating environmental factors [29]. One item from the questionnaire was not included in the PCA because it did not concern the perception of the environment, namely the *number of motorised vehicles in working order in household*. Analyses were repeated with that variable in the models, but it did not influence the outcome.

Factors with eigenvalues of ≥ 1.0 were extracted. Assuming that components may correlate with each other, oblimin rotation was performed to determine factor loadings. Factor scores were calculated using the Anderson-Rubin approach [29]. Given that the absolute values of factor scores are not informative *per se*, they were divided into tertiles for each factor. To ensure that all individual questions combined in factors by the PCA act in the same direction, bivariate relationships between the answers to each of the questions and physical activity categories and tertiles of walking were analysed using gamma statistics.

Independent associations between the physical environmental factor scores and the odds of being either in the middle or upper tertiles of walking, or the moderately or highly physically active category were performed in multinomial logistic regression models. Odds ratios and 95% confidence intervals (CI) between the tertiles of physical environmental factors and either tertiles of walking or categories of physical activity were analysed in relation to either the lowest walking tertile or the low physical activity category. For analyses of trend, the factor scores were entered in the analyses as continuous variables. The analyses were performed with and without adjustment for the potential confounders age, gender, self-perceived health, BMI, education, employment, marital status and smoking, and the highest category was used as the reference group for all independent variables in the models. The significance level was set to 5%. All statistical analyses were performed using SPSS version 14.0 (SPSS Inc., Chicago. IL).

## Results

The overall response rate was 59% resulting in a total of 1470 subjects. The responders did not differ from the Swedish population by age (p = 0.946), but the proportion of women was slightly higher among the responders than in general population (52.9% vs. 50.2%, p = 0.034).

The distribution of the total health enhancing physical activity and median amount of minutes spent walking across background characteristics of the sample are presented in Table 2.

**Table 2:** Categories of health enhancing physical activity and median weekly minutes of walking by sample characteristics

	N	Health enhancing physical activity			p^a^	Walking (Median)	p^b^
					
		Low (%)	Moderate (%)	High (%)			
Gender					0.001		0.020
Women	777	38.5	42.3	19.1		180.0	
Men	693	35.5	31.0	33.5		140.0	
Age					0.001		0.045
18–34	395	29.8	37.6	32.6		120.0	
35–54	566	36.5	40.4	23.1		150.0	
55–74	509	43.6	32.5	23.9		180.0	
BMI							
< 25.0	819	33.8	39.6	26.6	0.001	180.0	0.021
25.0–29.9	508	37.5	34.6	27.9		125.0	
≥ 30.0	118	58.9	29.5	11.6		140.0	
Education					0.001		0.534
College/university	443	38.9	42.1	19.0		175.0	
High school	632	32.0	38.3	29.7		150.0	
Other	77	35.2	35.2	29.6		180.0	
Basic school	318	45.4	26.8	27.8		165.0	
Employment status					0.001		0.094
Employed	880	34.3	39.4	26.4		150.0	
Student	126	25.4	43.4	31.1		105.0	
Retired	245	49.8	29.3	21.0		150.0	
Unemployed/unknown	219	41.5	31.5	27.0		210.0	
Marital status					0.110		0.712
Single	420	33.8	36.6	29.6		150.0	
Married/Partner	1046	38.4	37.1	24.5		150.0	
Smoking status					0.349		0.125
Never smoked	765	34.9	38.1	27.0		140.0	
Former smoker	398	40.7	33.6	25.3		180.0	
Current smoker	293	37.1	38.6	24.3		180.0	
Self-perceived health					0.001		0.040
Excellent	272	23.2	36.9	39.9		200.0	
Very good	399	31.7	40.5	27.8		140.0	
Good	478	40.3	38.6	21.1		177.5	
Satisfactory or poor	309	51.8	28.8	19.4		127.5	

Total	1470^c^	37.1	36.9	26.0		150.0	

^a ^Calculated by χ^2^-test

^b ^Calculated by Mann-Whitney test (two groups) or Kruskal-Wallis test (three or more groups)

^c^Total numbers may not be equal to 1470 and 100% due to missing data in a few variables

As a result of the PCA, four factors with eigenvalues ≥ 1.0 were retained. These factors were labelled as *degree of urbanisation*, *traffic intensity*, *opportunities and aesthetics *of the area and the *fear of crime *and explained 31.3%, 15.4%, 8.6% and 7.3% of the total variance, respectively (Table 3). The factors should be interpreted such that being in the lowest tertile of the factor *degree of urbanisation *means that a subject lives in the least urbanised area. Being in the lowest tertile of the factor *traffic intensity *means that the traffic intensity is lowest. Subjects being in the lowest tertile of the factor *opportunities and aesthetics *have rated their neighbourhood as having few interesting things to look at and few opportunities to be physically active. Those in the lowest tertile of the factor *fear of crime *perceive that there is little crime in their neighbourhood. No significant discrepancies between the direction of the association of the factors and the individual questions contributing to these factors were detected (data not shown).

**Table 3:** Factor loadings for the four factors selected by PCA, all loadings ≥ 0.4 are shown

	Environmental factors			
	
Questionnaire item	Degree of urbanisation	Traffic intensity	Aesthetics and opportunities	Fear of crime
There are sidewalks on most of the streets in my neighbourhood.	0.79			
The sidewalks in my neighbourhood are well maintained and not obstructed.	0.79			
Many shops are within walking distance of my home.	0.76			
There are many places to go within easy walking distance of my home.	0.76			
Places for cycling in and around my neighbourhood are well maintained and not obstructed.	0.74			
There are facilities to cycle in or near my neighbourhood.	0.66			
My neighbourhood has several free or low cost recreation facilities.	0.66		0.45	
It is less than a 10–15 min walk to a transit stop from my home.	0.61			
Main type of housing in the neighbourhood (House or Semi-detached house, 1–2 stories, 3–4 stories, 5 stories or higher).	0.58			
There is so much traffic on the streets that it makes it difficult or unpleasant to ride a bicycle.		0.82		
There is so much traffic on the streets that it makes it difficult or unpleasant to walk.		0.76		
There are many four-way intersections in my neighbourhood.		0.56		
There are many interesting things to look at while walking in my neighbourhood.			0.75	
I see many people being physically active in my neighbourhood.			0.72	
The crime rate in my neighbourhood makes it unsafe to go on walks during the day.				0.83
The crime rate in my neighbourhood makes it unsafe to go on walks at night.				0.74

### Walking

When the factor scores were entered as a continuous variable, being in the middle tertile of walking was positively associated with the factor *opportunities and aesthetics *(p for trend = 0.037) and inversely with the factor *fear of crime *(p for trend = 0.024) (Table 4). Subjects in the lowest tertile of the factor *degree of urbanisation *had a 0.66 (95% CI 0.47 – 0.98) lower odds and those in the lowest tertile of the factor *fear of crime *had a 1.84 (95% CI 1.28 – 2.64) higher odds of being in the middle tertile of walking.

**Table 4:** Environmental factors by tertiles of walking

	Middle tertile of walking (80–300 minutes per week)				Upper tertile of walking (> 300 minutes per week)			
	
Factors	Crude odds ratio (95% CI)	p^a^	Adjusted odds ratio^b ^(95% CI)	p^a^	Crude odds ratio (95% CI)	p^a^	Adjusted odds ratio^b^ (95% CI)	p^a^
Degree of Urbanisation		0.109		0.104		0.024		0.800
Lower tertile	0.77 (0.56–1.06)		0.66 (0.47–0.98)		0.64 (0.46–0.87)		0.65 (0.45–0.95)	
Middle tertile	0.95 (0.68–1.31)		0.93 (0.66–1.33)		0.89 (0.64–1.22)		0.97 (0.68–1.37)	
Upper tertile	1.00 (Reference)		1.00 (Reference)		1.00 (Reference)		1.00 (Reference)	
								
Traffic intensity		0.225		0.595		0.633		0.142
Lower tertile	1.21 (0.88–1.66)		1.17 (0.82–1.68)		0.87 (0.64–1.20)		0.85 (0.59–1.22)	
Middle tertile	1.27 (0.92–1.77)		1.17 (0.82–1.67)		1.02 (0.75–1.42)		1.02 (0.72–1.44)	
Upper tertile	1.00 (Reference)		1.00 (Reference)		1.00 (Reference)		1.00 (Reference)	
								
Opportunities and Aesthetics		0.689		0.037		0.009		0.010
Lower tertile	0.88 (0.64–1.21)		0.99 (0.70–1.42)		0.63 (0.46–0.87)		0.67 (0.47–0.95)	
Middle tertile	0.99 (0.71–1.37)		0.98 (0.69–1.40)		0.91 (0.66–1.26)		0.94 (0.69–1.34)	
Upper tertile	1.00 (Reference)		1.00 (Reference)		1.00 (Reference)		1.00 (Reference)	
								
Fear of crime		0.004		0.024		0.122		0.434
Lower tertile	1.67 (1.21–2.29)		1.84 (1.28–2.64)		1.36 (0.98–1.88)		1.64 (1.14–2.36)	
Middle tertile	1.08 (0.78–1.50)		1.07 (0.75–1.52)		1.14 (0.84–1.57)		1.16 (0.82–1.63)	
Upper tertile	1.00 (Reference)		1.00 (Reference)		1.00 (Reference)		1.00 (Reference)	

^a^P-values for linear trends, which were examined by introducing factor scores as continuous variables

^b^Adjusted for age, gender, self-perceived health, BMI, education, employment, marital status, smoking and variables in the table

The odds of being in the upper tertile of walking increased with increasing factor score for the factor *opportunities and aesthetics *(p for trend = 0.010) (Table 4). Subjects in the lowest tertile of the factors *degree of urbanisation *had a lower odds 0.65 (95% CI 0.45 – 0.95) of being in the upper tertile of walking. The same were seen for the factor *opportunities and aesthetics *were those in the lowest tertile had a lower odds 0.67 (95% CI 0.47 – 0.95) of being in the upper tertile of walking. Those in the lowest tertile of the factor *fear of crime *had a increased odds 1.64 (95% CI 1.14 – 2.36) of being in the upper tertile of walking.

### Health enhancing physical activity

No statistically significant associations between the environmental factors and the odds of being in the moderately physically active category were found (Table 5). There was however a borderline significant inverse trend between being in the moderate category and the factor *degree of urbanisation *(p for trend = 0.086) when the factor scores was entered as a continuous variable in the model.

**Table 5:** Environmental factors by categories (moderate and high) of health enhancing physical activity

	Moderate category				High category			
	
Factors	Crude odds ratio (95% CI)	p^a^	Adjusted odds ratio^b ^(95% CI)	p^a^	Crude odds ratio (95% CI)	p^a^	Adjusted odds ratio^b^ (95% CI)	p^a^
Degree of Urbanisation		0.705		0.086		<0.001		<0.001
Lower tertile	1.06 (0.78–1.44)		1.36 (0.94–1.71)		1.53 (1.09–2.16)		2.01 (1.32–3.05)	
Middle tertile	1.08 (0.79–1.47)		1.27 (0.89–1.77)		1.28 (0.90–1.81)		1.52 (1.02–2.25)	
Upper tertile	1.00 (Reference)		1.00 (Reference)		1.00 (Reference)		1.00 (Reference)	
								
Traffic Intensity		0.102		0.687		0.005		0.472
Lower tertile	1.09 (0.79–1.49)		0.91 (0.64–1.31)		1.43 (1.02–2.01)		1.02 (0.68–1.51)	
Middle tertile	1.21 (0.89–1.66)		1.09 (0.67–1.38)		1.18 (0.83–1.68)		0.96 (0.65–1.42)	
Upper tertile	1.00 (Reference)		1.00 (Reference)		1.00 (Reference)		1.00 (Reference)	
								
Opportunities and Aesthetics		0.010		0.218		0.097		0.049
Lower tertile	0.78 (0.57–1.06)		0.92 (0.65–1.31)		0.83 (0.59–1.17)		0.77 (0.52–1.14)	
Middle tertile	1.04 (0.76–1.41)		1.13 (0.80–1.59)		0.80 (0.57–1.13)		0.85 (0.58–1.26)	
Upper tertile	1.00 (Reference)		1.00 (Reference)		1.00 (Reference)		1.00 (Reference)	
								
Fear of crime		0.200		0.843		0.040		0.963
Lower tertile	1.10 (0.80–1.50)		1.02 (0.71–1.47)		1.38 (0.99–1.94)		0.99 (0.67–1.48)	
Middle tertile	1.21 (0.89–1.66)		1.17 (0.83–1.66)		1.08 (0.76–1.53)		0.97 (0.65–1.43)	
Upper tertile	1.00 (Reference)		1.00 (Reference)		1.00 (Reference)		1.00 (Reference)	

^a^P-values for linear trends, which were examined by introducing factor scores as continuous variables

^b^Adjusted for age, gender, self-perceived health, BMI, education, employment, marital status, smoking and variables in the table

A significant inverse trend between the odds of being in the high category and *degree of urbanisation *(p for trend < 0.001) was found (Table 5). Participants living in the least and moderately urbanised areas had 2.01 (95% CI 1.32 – 3.05) and 1.52 (95% CI 1.02 – 2.25) times higher odds of reaching the highly physically active category than those living in the most urbanised areas independently of other studied characteristics. There was also a significant positive trend between the highly physically active category and *opportunities and aesthetics *(p for trend = 0.049).

## Discussion

This study examined the association between features of the perceived neighbourhood environment and self reported levels of walking and total health enhancing physical activity in a nationally representative sample of Swedish adults. The *degree of urbanisation *was positively associated with walking. Furthermore, participants who perceived their environment as aesthetically pleasing and providing good opportunities to be physically active and who felt safe in their neighbourhood had the highest odds of being in the middle and upper tertile of walking.

### Walking

Our factor *degree of urbanisation *is similar to what others have termed the walkability of a neighbourhood, e.g. high residential density, a diverse mix of land use and high road connectivity [8,15,30-32]. A highly walkable neighbourhood has consistently been shown to facilitate walking among different populations. Since the factor *degree of urbanisation *turned out to be similar to the concept of walkability the positive association with tertiles of walking was expected, indicating a relatively supportive environment for walking in Swedish urbanised areas.

In the present study, as in many others [7,8,16,33], neighbourhoods which were perceived as aesthetically pleasing and offering recreational opportunities were also neighbourhoods where people were more likely to report high levels of walking.

Subjects who perceived their neighbourhood as safe from crime were more likely to be classified as being in the moderate or high tertile of walking. This relationship is similar to that found by Bennett *et al *[34] who found that women who did not feel safe outdoors at night-time took some 1000 steps fewer (assessed by pedometers) than those that felt safe. Our study suggests that this association is not confounded by sex. However, the literature on crime and walking is quite inconsistent across studies [8,16,35].

### Health enhancing physical activity

While the results for walking were in line with results from other studies, this was not the case for levels of total health enhancing physical activity. A borderline statistically significant trend (p = 0.086) between the odds of being in the moderately physically active category and an inverse trend between highly physically active category and the degree of urbanisation (p < 0.001), was seen. These findings are in the opposite direction to results from other studies [14,30,36-40]. We know from previous research on the same population that walking is a big component of their total health enhancing physical activity [17]. Therefore, it had been assumed that the association between walking and the environmental factors would have been similar to the associations between total health enhancing physical activity and the environmental factors. This was not the case. One reason for this may be that the subjects in the highly urbanised category live in neighbourhoods that are supportive for walking, but not for physical activity of a higher intensity. And at the same time, subjects from the least urbanised category live in neighbourhoods that are not supportive for walking but support other kinds of physical activity such as gardening or recreational physical activity. Another reason could be that those living in urbanised areas are not accumulating sufficient amounts of physical activity within their own neighbourhood, but rather at other places e.g. at their workplace, during transportation or at fitness centers.

None of the factors *intensity of traffic*, or *fear of crime *was associated with categories of health enhancing physical activity. The impact of traffic on physical activity may vary by the purpose of the physical activity (i.e. for transport or for recreation), which was not captured in the present study.

In general, neighbourhoods perceived to be aesthetically pleasing and offering recreational opportunities are also neighbourhoods where people tend to report high levels of physical activity [7,8,33]. In our study we could see a positive trend (p = 0.049) between the degree of *opportunities and aesthetics *and total health enhancing physical activity. The point estimate, OR = 0.77, 95% Confidence Intervall: 0.52 – 1.14, although not statistically significant, indicates some degree of clinical relevance in the finding. This needs to be further investigated with regards to physical activity for various purposes.

Crime and fear of crime have shown inconsistent associations with physical activity [8], and better measures may be required to clarify the impact of crime on total health enhancing physical activity. It may be that crime or fear of crime only has influence over particular physical activity behaviours such as walking for recreation, while it has a limited influence over other behaviours. Studies to date have not assessed how people manage fear of crime, such as walking with others. This aspect should be included in future studies [41].

### Strengths and limitations

The use of a national sample, and the adjustment for many potential confounders, are among this study's strengths. The response rate was close to 60% which is comparable to studies of similar design conducted in Sweden[42,43]. Moreover, the study sample was fairly representative of the adult population of Sweden. The slight overrepresentation of women in the study sample is probably of little relevance for the main results.

Although the short version of the IPAQ is suitable for assessing health enhancing physical activity on a population level, and gives a fairly good approximation of the total health enhancing physical activity, it is not designed to assess the physical environment's influence on physical activity in different domains. As seen in our study, the same feature in the physical environment can be related to various physical activities in different ways. To advance understanding, a more specific physical activity measure is needed. The long version of the IPAQ is designed for this purpose . It asks about domain-specific physical activity in four different domains; during transportation, domestic and household chores, at work and during leisure time. Using the long version of IPAQ could provide more precise data on the physical environment's influence on domain-specific physical activity. This is an important area for future research, especially since the results from our study indicate a differential effect of the environment on different domains of physical activity.

## Conclusion

Walking was positively associated with the factors *degree of urbanisation *and *opportunities and aesthetics *and negatively to *fear of crime *while total health enhancing physical activity was negatively associated with the *degree of urbanisation*. These findings suggest the neighbourhood environment could affect different aspects of physical activity differently. Walking is an easy mode of physical activity, accessible to almost everyone, and increasing levels have been shown to confer health benefits. Thus interventions to increase walking in different societies must be of great concern. The results of this study serve to highlight the importance of assessing both total physical activity as well as its components.

## Competing interests

The authors declare that they have no competing interests.

## Authors' contributions

MS: Is a member of the international IPAQ Core Group, who initiated the International Prevalence Study (IPS), and he also initiated and lead the national Swedish study. JFS: Is a member of the international IPAQ core group and responsible for the international IPS environmental study. PB: Conducted the IPS fieldwork in Sweden and wrote the first draft of the manuscript. AMG: Performed the statistical analyses and drafted the manuscript. JFS, MH and MS all drafted the manuscript. All authors read and approved the final manuscript.
